# Epidemiology, Disease Severity and Outcome of Severe Acute Respiratory Syndrome Coronavirus 2 and Influenza Viruses Coinfection Seen at Egypt Integrated Acute Respiratory Infections Surveillance, 2020–2022

**DOI:** 10.1155/2022/7497500

**Published:** 2022-11-17

**Authors:** Manal Fahim, Wael H. Roshdy, Ola Deghedy, Reham Kamel, Amel Naguib, Shymaa Showky, Nancy Elguindy, Mohammad Abdel Fattah, Salma Afifi, Amira Mohsen, Amr Kandeel, Khaled Abdelghaffar

**Affiliations:** ^1^Department of Epidemiology and Surveillance, Preventive Sector, Ministry of Health and Population, 3 Magles El Shaab Street, Kasr Alainy, Cairo, Egypt; ^2^Central Public Health Laboratory, Ministry of Health and Population, Elsheikh Rehan Street, Cairo, Egypt; ^3^Preventive Sector, Ministry of Health and Population, 3 Magles ElShaab Street, Kasr Alainy, Cairo, Egypt; ^4^Consultant for Ministry of Health and Population, 3 Magles ElShaab Street, Kasr Alainy, Cairo, Egypt; ^5^World Health Organization, Egypt Country Office, 3 Magles ElShaab Street, Kasr Alainy, Cairo, Egypt; ^6^Ministry of Health and Population, 3 Magles ElShaab Street, Kasr Alainy, Cairo, Egypt

## Abstract

**Background:**

Cocirculation of influenza (Flu) and severe acute respiratory syndrome coronavirus 2 (SARS-CoV-2) (SARS-CoV-2/Flu) represent a public health concern as it may worsen the severity and increase fatality from coronavirus disease 2019. An increase in the number of patients with coinfection was recently reported. We studied epidemiology, severity, and outcome of patients with SARS-CoV-2/Flu coinfection seen at Egypt's integrated acute respiratory infections surveillance to better describe disease impact and guide effective preventive measures.

**Methods:**

The first two outpatients were seen daily, and every fifth patient admitted to 19 sentinel hospitals with respiratory symptoms was enrolled. Patients were interviewed using a standardized questionnaire and provided nasopharyngeal swabs to be tested for SARS-CoV-2 and influenza by real-time polymerase chain reaction at the central laboratory. Data from all patients with coinfection were obtained, and descriptive data analysis was performed for patients' demographics, clinical course, and outcome.

**Results:**

The total number of patients enrolled between January 2020 and April 2022 was 18,160 and 6,453 (35.5%) tested positive for viruses, including 52 (0.8%) coinfection. Of them, 36 (69.2%) were coinfected with Flu A/H3, 9 (17.3%) Flu-B, and 7 (13.5%) Flu A/H1. Patients' mean age was 33.2 ± 21, 55.8% were males, and 20 (38.5%) were hospitalized, with mean hospital days 6.7 ± 6. At the hospital, 14 (70.0%) developed pneumonia, 6 (30.0%) ICU admitted, and 4 (20.0%) died. The hospitalization rate among patients coinfected with Flu-B and Flu A/H3 was 55.6 and 41.7%, with mean hospital days (8.0 ± 6 and 6.4 ± 6), pneumonia infection (40.0 and 80.0%), ICU admission (40.0 and 26.7%), and death (20.0% for both), while no patients hospitalized with A/H1.

**Conclusions:**

The recent increase in the number of SARS-CoV-2/Flu coinfections was identified in Egypt. The disease could have a severe course and high fatality, especially in those coinfected with Flu-B and Flu A/H3. Monitoring disease severity and impact is required to guide preventive strategy.

## 1. Introductions

Cocirculation of the influenza virus (Flu) and severe acute respiratory syndrome coronavirus 2 (SARS-CoV-2) may worsen the severity and increase fatality from coronavirus disease 2019 (COVID-19) as a result of misdiagnosis or delay in patient treatment [1]. Recently, an increase in the number of patients with Flu and SARS-CoV-2 (SARS-CoV-2/Flu) coinfection has been reported in many countries [2, 3]. Although the number of cases reported globally is comparatively small, yet underreporting is likely because of the high rates of singular infections reported [4].

During the first year of the COVID-19 pandemic, influenza viral infections reported to the World Health Organization have dropped to minimal levels [5]. However, influenza reemerged at the end of 2021 [6]. Researchers suggested that the decreased circulation of influenza viruses could be related to the nonpharmaceutical interventions (NFI) used to control COVID-19. In the event that the NFI returns to normal after such low influenza activity, the global population might lose herd immunity to the disease, which could bring about a double epidemic of COVID-19 and influenza [7].

Few studies described the epidemiologic characteristics, disease severity, and outcome of patients with SARS-CoV-2/Flu coinfection [4, 8, 9]. One reason could be that comprehensive surveillance systems with well-established laboratory capacity for SARS-CoV-2 and influenza molecular testing are not readily available especially in developing countries [10].

Egypt MoHP introduced influenza surveillance as early as 1999 through the establishment of Influenza-like illness (ILI) sentinel sites whereas surveillance for severe acute respiratory infections (SARI) was initiated in Egypt in November 2007 at eight governmental infectious diseases and chest hospitals with wide geographical coverage [11]. The surveillance system is maintained by the acute respiratory diseases unit under the Department of Epidemiology and Surveillance within the MoHP preventive sector. As of 2016, ILI, SARI, and pneumonia were combined into “the integrated acute respiratory infection surveillance.” Currently, the surveillance system in Egypt is quite robust, with 27 sentinel sites within 19 MoHP hospitals covering 13 governorates all over the country, as well as the regional laboratories [12]. SARS-CoV-2 was introduced in the surveillance at the beginning of the COVID-19 pandemic in addition to influenza viruses [12].

The first case of SARS-CoV-2/Flu coinfection in Egypt was reported from an ILI site three months after the first COVID-19 case was detected in mid-February 2020 [13]. Reports from the surveillance indicated that more cases of coinfection are identified as the pandemic progressed.

## 2. Study Objectives

This study aims to better describe the epidemiology, disease severity, and outcome of SARS-CoV-2/Flu coinfection to guide the development of effective preventive and control measures including case management and vaccination policy.

## 3. Methods

### 3.1. Surveillance Methods

Surveillance targets all SARI patients hospitalized with acute respiratory symptoms. Patients are enrolled according to WHO SARI standard case definition (fever ≥ 38°C, history of sudden onset of fever, and cough within the last 10 days) with no age exclusion. If the patient has confirmed pneumonia by X-ray, a case report form is administered to collect patient data. All patients hospitalized with SARI and pneumonia are logged in a logbook and are followed prospectively until discharge or death or transfer to another hospital.

During the COVID-19, pandemic staff and resources were diverted to support the pandemic response. To compensate for the critical shortage of surveillance teams and laboratory capacity, surveillance methods were modified to enroll every fifth hospitalized patient with SARI.

Additionally, patients with ILI symptoms visiting the outpatient clinics in the sentinel sites are included. The WHO ILI standard case definition (fever ≥ 38°C, history of sudden onset of fever, and cough within the last 10 days) is used to identify the first two ILI patients seen every day at the ILI sites.

### 3.2. Study Population

The study included all patients with SARS-CoV-2/Flu coinfection presented to the sentinel sites between January 2020 and April 2022 who were enrolled with no age exclusion [5].

### 3.3. Study Tool

Enrolled patients are interviewed using standardized data collection tools adapted from WHO's Global Epidemiological Surveillance Standards for Influenza used to collect patients' demographics, clinical pictures, discharge diagnoses, and outcome information. As soon as the COVID-19 pandemic emerged, data collection tools with all variables required for weekly reporting on COVID-19 at the global level were included. Case definitions of COVID-19 were developed, regularly revised, and distributed to hospitals.

### 3.4. Sampling Technique

Considering 1% prevalence of SARS-CoV-2/Flu coinfection as reported by many studies^1–3, 7^ and with a study design value of 2, a minimum sample size of 31 subjects will provide 5% confidence and precision of 20%. The sample size was calculated using OpenEpi, Version 3, an open-source calculator [14].

Since the percentage of SARS-CoV-2/Flu coinfection globally is still low ranging from (0.4–4.5%) [6], all patients with SARS-CoV-2/Flu coinfection from January 2020 to April 2022 were included in this study in order to describe the disease's clinical picture, severity, and outcome.

### 3.5. Laboratory Investigations

After consent, patients were asked to provide two throat and nasopharyngeal swabs for laboratory testing. One was tested immediately for SARS-CoV-2 to avoid delay in diagnosis, while the other was maintained in viral transport media, stored in a nitrogen tank at –80°C, and shipped on a weekly basis to the Central Public Health Laboratory (CPHL) in Cairo for influenza type and subtype testing.

Specimens were tested for SARS-CoV-2 by nucleic acid extraction using the Chemagic 360 Instrument (PerkinElmer Inc). SARS-CoV-2 RNA (ORF1ab) was detected using a VIASURE SARS-CoV-2 real-time polymerase chain reaction (RT-PCR) detection kit (Certest Biotec SL). The RT-PCR runs were performed in triplicate, and according to the manufacturer's recommendations, the samples were confirmed for SARS-CoV-2 using a Cobas 6800 system (Roche Holding AG). Influenza type and subtype were tested by RT-PCR using the WHO guidelines [15].

### 3.6. Data Analysis

Surveillance data are entered using a web-based application developed by the Ministry of Health and Population (MoHP) to create comprehensive influenza and SARS-CoV-2 database. The database was accessed to obtain data of all patients with SARS-CoV-2/Flu coinfection. Descriptive data analysis by time, person, and place was performed with frequencies, percentages, means, and standard deviations using Epi info7 software. Demographic and clinical data of patients were compared by influenza subtype to describe disease severity and outcome of coinfection with different influenza virus subtypes.

## 4. Results

**Figure 1:**
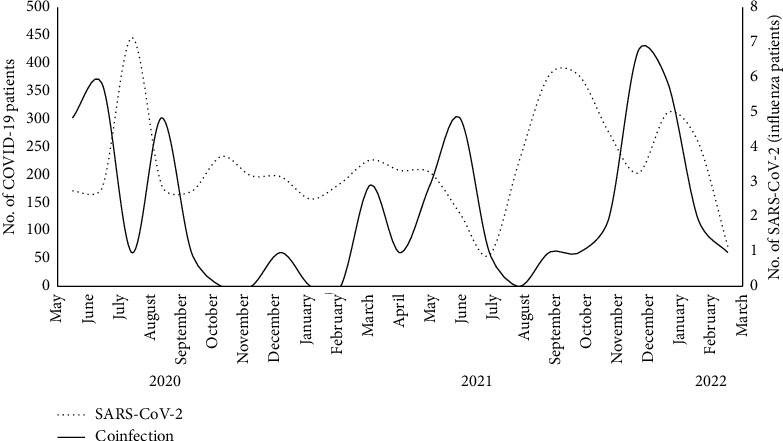
Montlthy distribution of COVID-19 and SARS-CoV-2/influenza co-infection, Egypt integrated acute respiratory surveillance, 2020–2022.

Between January 2020 and April 2022, 18,160 patients were reported by the 27 surveillance sites including 10,237 (56.4%) by the SARI and pneumonia and 7,923 (43.6%) by the ILI sites. Among all patients, 6,453 (35.5%) were positive for one or more of the tested viruses, with SARI (45.7%) having a higher rate of positivity than ILI patients (22.4%). Among the 6,453 positive patients, 52 (0.8%) had SARS-CoV-2/Flu coinfection, with a higher rate found in ILI patients than in SARI patients (1.8 vs. 0.4%, *p* < 0.001). The mean age of coinfected patients was 33.2 ± 21, most of them 37 (71.2%) were middle-aged (15–64 years), and 55.8% were males. A higher number of patients with coinfection 34 (65.4%) was reported from Upper Egypt, during the spring to summer months 31 (59.6%), and most patients 32 (61.5%) did not require hospital admission and were treated at home (Table 1). The increase in the number of coinfections occurred after the increase in the number of COVID-19 cases reported to the sentinel surveillance (Figure 1).

Among the 52 patients with coinfection, 20 (38.5%) were hospitalized, including 6 (30.0%) who had comorbidities. Hospitalized patients were significantly older than nonhospitalized (42.1 ± 21 vs. 27.6 ± 20 years of age). They experienced severe disease courses in terms of developing pneumonia 14 (70.0%), ICU admission 6 (30.0%), length of hospital stay (6.7 ± 6 days), and case fatality rate 4 (20.0%) (Table 1). Mortality was highest among patients ≥65 years of age (33.3%), followed by 28.6% in 35–64 years and 11.1% in the 15–34 years group, while no fatalities were reported in the <15 years of age. Of the four patients with coinfection who died at the hospital, 3 (75.0%) had comorbidities.

**Figure 2:**
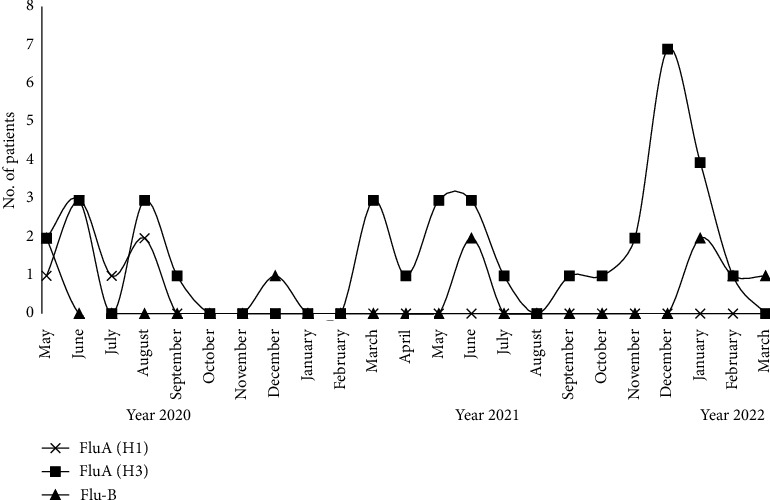
Monthly distribution of SARS-CoV-2 and influenza co-infection by influenza subtype, Egypt integrated acute respiratory surveillance, 2020–2022.

Of all cases of SARS-CoV-2/Flu coinfection, 36 (69.2%) were coinfected with Flu A/H3, 9 (17.3%) with Flu-B, and 7 (13.5%) with Flu A/H1. Coinfections with Flu-B and Flu A/H3 were reported during the pandemic years 2021 and 2022 and occurred mainly in the middle-aged (mean age 29.8 ± 20 and 30.7.4 ± 21, respectively) whereas coinfection with Flu A/H1 occurred early in the pandemic in the summer of 2020, in the elderly (mean age 50.3 ± 22). Patients admitted with SARS-CoV-2/Flu-B (*n* = 5) and Flu A/H3 coinfections (*n* = 15) had severe disease courses in terms of higher hospitalization rate (55.6 and 41.7%), longer hospital stay (mean hospital days 8.0 ± 8 and 6.4 ± 6), higher rate of pneumonia (40.0 and 80.0%), ICU admission (40.0 and 60.0%), and case fatality rate (20.0% for both), respectively, compared to patients coinfected with A/H1 who did not require hospitalization (Table 2, Figure 2). A peak of coinfection with A/H3 occurred during the 2021–2022 influenza season where 27 of the 33 coinfections reported (81.8%) were caused by the A/H3 influenza virus (Figure 2).

## 5. Discussion

SARS-CoV-2, influenza, and SARS-CoV-2/Flu coinfection have created a unique challenge for healthcare workers as the two viruses have similar clinical presentation and transmission characteristics. There is currently a rise in the number of SARS-CoV-2/Flu coinfection globally, while no accurate estimate of the disease burden is available due to the limited number of coinfection cases reported in the literature [2, 4]. A knowledge gap concerning disease morbidity and mortality created a need for further studies to assist clinicians in managing cases effectively and decision-makers in estimating disease burden. This study reports a reasonable number of patients with SARS-CoV-2/Flu coinfection, allowing for a more comprehensive understanding of the disease's epidemiology and severity.

Early in the COVID-19 pandemic, many countries, including Egypt, reported concomitant infection with SARS-CoV-2 and influenza [2, 8, 9, 13]. Studies reported different percentages of coinfection among laboratory-confirmed cases ranging from a few cases to 57.3% of COVID-19 patients admitted to a single center in China [9, 16]. This study identified a low percentage of patients with coinfection among patients presented to Egypt Integrated surveillance; however, underreporting is likely as only one-fifth of patients admitted to the sentinel sites are tested for influenza due to the surge of patients with SARI during the pandemic. The discrepancy in the prevalence of SARS-CoV-2/Flu coinfection reported by other studies could be explained by the different lab testing protocols and available kits, patient selection methods, and incidence of SARS-CoV-2 and influenza in different countries and periods [16]. Standard surveillance methods and systematic laboratory testing for SARS-CoV-2 and influenza are recommended to accurately estimate the disease burden.

This study indicated that the largest proportion of patients with SARS-CoV-2/Flu co-infection occurs in the middle age group; in addition, most of the patients had experienced mild symptoms and were treated at home. This could reflect characteristics of influenza viral infection that affects mainly young ages and does not usually require hospitalization [17].

The high proportion of coinfections found in the Upper Egypt region in this study where COVID-19 rates are high, together with the increase in coinfections after COVID-19 peaks, suggesting a relationship between coinfections and COVID-19.

This study demonstrated an overlap of the epidemiologic and clinical courses attributable to either SARS-CoV-2 or influenza infection which poses a challenge for clinicians in diagnosis and case management. However, the severity of the disease course among hospitalized patients and the presence of comorbidity found in this study could help clinicians to suspect coinfection and manage cases accordingly.

Surveillance data from England suggested that patients coinfected with SARS-CoV-2 and influenza could exhibit more severe disease outcomes [2]. This study identified severe disease courses for hospitalized patients in terms of pneumonia infection, ICU admission, days of hospital stay, and case fatality rate similar to other studies [1, 2, 18, 19]. However, almost 2/3 of the studied patients with coinfection did not require hospitalization and experienced mild symptoms. The reasons could be the younger ages and the absence of risk factors for severe outcomes in those patients. This could indicate that coinfection could cause mild disease if occurs in young patients with no risk factors, while it may lead to a severe outcome in the elderly with comorbidities.

The in-hospital fatality rate among SARS-CoV-2/Flu coinfection is controversial, a study conducted in Bangladesh did not report any deaths among coinfected SARI case-patients where patients were more likely to be referred to other healthcare facilities or lost to follow-up after hospital discharge [20]. Other studies conducted in different countries reported an increase or decrease in fatality rates compared to SARS-CoV-2 infection [2, 21–23]. This study reported higher rates of fatality among hospitalized patients with coinfection compared to rates of fatality from SARS-CoV-2 and influenza infections in Egypt [17]. The severity of the disease course and high fatality rates reported in this study could be related to the small number of hospitalized patients with coinfection, the high prevalence of comorbidities among them, or delay in influenza testing which could lead to delay in the effective case management.

The study indicated that routine SARS-CoV-2 screening is not sufficient to exclude the possibility of coinfection. The results of this study support the idea that systematic testing for influenza coinfection in COVID-19 patients should be conducted especially among hospitalized to detect the disease early and effectively manage it.

Surveillance indicated that the number of patients with coinfection has peaked with the peak of the 2021–2022 influenza season in Egypt. This could support WHO recommendations for countries to prepare for the cocirculation of influenza during the influenza seasons and could guide clinicians in case diagnosis and management [5].

Several studies reported that the higher severity and case fatality of SARS-CoV-2/Flu coinfection could be associated with the influenza subtype including Flu-B and A/H1 subtypes [16, 23]. This study reported more severe disease course and fatality rates among patients coinfected with subtypes B and A/H3. Previously, there was a conventional concept that Flu A causes more severe illnesses than Flu-B subtype; however, a recent study claimed that the clinical severity of Flu-B is similar to Flu A [24]. Study results could support considering the use of quadrivalent influenza vaccines that contain both circulating lineages of B viruses.

### 5.1. Study Limitations

The study is subject to at least two limitations, first is the need for a larger number of patients to develop a solid conclusion and second is that enrollment of one-fifth of hospitalized SARI patients could lead to underreporting of the disease and reduce estimates of its burden.

## 6. Conclusions

A recent increase in the number of SARS-CoV-2/Flu coinfection was identified in Egypt especially during SARS-CoV-2 and influenza high activity seasons. The study indicated that the disease is potentially severe and could lead to high mortality, especially in hospitalized older patients with comorbidities. Coinfection of SARS-CoV-2 with Flu-B and with Flu A/H3 had a more severe course and higher fatality than Flu A/H1. Clinicians should suspect coinfection in risky groups and monitor surveillance closely to ensure accurate diagnosis and effective case management. Surveillance with standard systematic SARS-CoV-2 and influenza testing is crucial for monitoring both virus activities and describing disease burden.

## Figures and Tables

**Table 1:** Epidemiology, disease severity and outcome of the SARS-CoV-2 and influenza co-infection, Egypt integrated acute respiratory surveillance, 2020–2022.

Characteristics	Number of co infection cases (*n* = 52)	Percent
Mean age ± SD±±±	33.2 ± 21	
Age groups		
Less than 5	4	7.7
5–14	6	11.5
15–34	20	38.5
35–64	17	32.7
≥65	5	9.6
Gender		
Male	29	55.8
Female	23	44.2
Region		
Upper Egypt	34	65.4
Lower Egypt	10	19.2
Urban Gov	8	15.4
Season		
Autumn	5	9.6
Winter	16	30.8
Spring	13	25.0
Summer	18	34.6
Healthcare type		
Home treatment	32	61.5
Hospitalized	20	38.5
With comorbidities	6	30.0
Pneumonia	14	70.0
ICU admitted	6	30.0
Ventilated	1	5.0
Mean hospital days ± SD (range)	6.7 ± 6	Range [1–19]
Died at hospital	4	20.0

**Table 2:** A comparison of epidemiology, severity and outcome of SARS-CoV-2 and influenza co-infection patients by influenza subtype, Egypt integrated acute respiratory surveillance, 2020–2022.

Characteristics	SARS-CoV-2 and Flu A/H3 co-infection (*n* = 36)		SARS-CoV-2 and Flu-B (*n* = 9) co-infection		SARS-CoV-2 and Flu A/H1 (*n* = 7) co-infection	
	Number	Percent	Number	Percent	Number	Percent
Mean age years ± SD	30.7 ± 21		29.8 ± 21		50.3 ± 22	
Male gender	19	52.8	7	77.8	3	42.9
Region						
Upper Egypt	24	66.7	5	55.6	5	71.4
Lower Egypt	7	19.4	1	11.1	2	28.6
Urban Gov	5	13.9	3	33.3	0	0.0
Season						
Autumn	5	13.9	0	0.0	0	0.0
Winter	12	33.3	4	44.4	0	0.0
Spring	9	25.0	3	33.3	1	14.3
Summer	10	27.8	2	22.2	6	85.7
Severity						
Hospitalized	15	41.7	4	55.6	0	0.0
With comorbidity	4	26.7	2	40.0	NA	NA
Pneumonia	12	80.0	2	40.0	NA	NA
ICU admitted	4	26.7	2	40.0	NA	NA
Mean hospital days ± SD	6.4 ± 6		8.0 ± 8		NA	NA
Died at hospital	3	20.0	1	20.0	NA	NA

## Data Availability

The datasets generated and/or analyzed during the current study are not publicly available due to privacy restrictions but are available from the Egyptian Ministry of Health and Population upon reasonable request.
